# Local Recurrence and Oncological Outcomes After Transanal Total Mesorectal Excision With Intersphincteric Resection for Lower Rectal Cancer: A Nationwide Multicenter Cohort Study (Ta‐Ta‐Mi Study)

**DOI:** 10.1002/ags3.70304

**Published:** 2026-10-31

**Authors:** Kan Tanabe, Shinichiro Mori, Kenji Baba, Takeru Matsuda, Ichiro Takemasa, Koji Okabayashi, Chikayoshi Tani, Tatsuya Manabe, Yoshihiro Kakeji, Takao Ohtsuka, Seiichiro Yamamoto, Takeshi Naitoh, Ta‐Ta‐Mi Study Group Collaborative of the Japan Society of Laparoscopic Colorectal Surgery

**Affiliations:** ^1^ Department of Digestive Surgery Kagoshima University Graduate School of Medical Sciences Kagoshima Kagoshima Japan; ^2^ Division of Gastrointestinal Surgery, Department of Surgery Kobe University Graduate School of Medicine Kobe Japan; ^3^ Gastroenterological Surgery Osaka International Medical & Science Center Osaka Japan; ^4^ Department of Surgery Keio University School of Medicine Tokyo Japan; ^5^ Division of Gastrointestinal Surgery, Department of Surgery Asahikawa Medical University Asahikawa Japan; ^6^ Department of Surgery, Faculty of Medicine Saga University Saga Japan; ^7^ Department of Gastroenterological Surgery Tokai University School of Medicine Isehara Japan; ^8^ Department of Lower Gastrointestinal Surgery Kitasato University School of Medicine Sagamihara Japan

**Keywords:** intersphincteric resection, neoplasm recurrence, rectal neoplasms, risk factors, transanal total mesorectal excision

## Abstract

**Aim:**

Transanal total mesorectal excision (TaTME) combined with intersphincteric resection (ISR) has emerged as a sphincter‐preserving surgical option for very low rectal cancer. However, multicenter data evaluating oncological outcomes and risk factors for local recurrence (LR) following TaTME with ISR remain limited. This study aimed to evaluate oncological outcomes and identify risk factors for LR in patients undergoing TaTME with ISR.

**Methods:**

This multicenter retrospective cohort study included patients who underwent TaTME with ISR for lower rectal cancer at 26 institutions participating in the Japan Society for Laparoscopic Colorectal Surgery. Oncological outcomes and clinicopathological factors associated with LR were analyzed. Multivariable analysis was performed to identify independent risk factors for LR.

**Results:**

A total of 224 patients were included. The 3‐year LR, recurrence‐free survival (RFS), and overall survival (OS) rates were 7.3%, 79.5%, and 98.1%, respectively. Multivariable analysis identified circumferential resection margin (CRM)/radial margin (RM) positivity as the key independent risk factor for LR. In contrast, pathological features, including histopathological classification, lymphatic invasion, vascular invasion, perineural invasion, and tumor budding, reflected tumor biological aggressiveness rather than surgical performance. A biological aggressiveness score demonstrated a stepwise increase in LR risk according to the number of adverse pathological factors.

**Conclusion:**

TaTME combined with ISR is a feasible sphincter‐preserving option for lower rectal cancer, with acceptable oncological outcomes in a nationwide multicenter cohort. Both surgical factors, particularly margin status, and tumor biological characteristics may contribute to LR risk. Careful margin assessment and close postoperative surveillance are essential for optimizing oncological outcomes.

## Graphical abstract

The 3‐year local recurrence (LR), recurrence‐free survival, and overall survival rates were 7.3%, 79.5%, and 98.1%, respectively. Multivariable analysis identified circumferential resection margin (CRM)/radial margin (RM) positivity as an independent risk factor for LR. An exploratory biologic aggressiveness score demonstrated a stepwise increase in the 3‐year cumulative incidence of LR with an increasing number of adverse pathological factors. TaTME combined with ISR may be a feasible sphincter‐preserving option for selected patients with lower rectal cancer, with acceptable oncological outcomes.

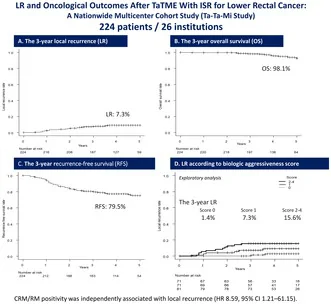

## 1 Introduction

Transanal total mesorectal excision (TaTME), first reported by Sylla et al. in 2010 [1], has emerged as a minimally invasive approach for mid‐ and low‐rectal cancer. By providing a direct transanal view of the distal mesorectum, TaTME facilitates precise dissection in the deep pelvis and may improve visualization of the mesorectal plane, particularly in patients with a narrow pelvis, bulky mesorectum, or tumors located close to the anal verge [2, 3].

Despite these potential advantages, concerns regarding the oncological safety of TaTME were raised following reports from Norway describing unexpectedly high local recurrence (LR) rates with a multifocal recurrence pattern during the early implementation period [4]. Subsequent analyses from international registries and large multicenter cohort studies have demonstrated LR rates comparable to those observed after conventional total mesorectal excision (TME), suggesting that TaTME can achieve acceptable oncological outcomes in a multicenter setting [5, 6].

Recently, the nationwide Japanese Ta‐Ta‐Mi study evaluated oncological outcomes after TaTME in 706 patients treated across 26 institutions and reported a 3‐year cumulative LR rate of 6.82% [7]. That study also identified several risk factors for LR, including tumor height from the anal verge (AV), pathological stage, perineural invasion, and positive resection margins, suggesting that both tumor biology and surgical factors contribute to recurrence after TaTME.

Intersphincteric resection (ISR), first described by Schiessel [8], is a sphincter‐preserving procedure for very low rectal cancer and an alternative to abdominoperineal resection (APR). ISR is considered one of the most technically demanding sphincter‐preserving procedures. Because ISR requires precise dissection along the intersphincteric plane in the deep pelvis, the technical advantages of TaTME may be particularly beneficial for this procedure [9, 10]. However, ISR itself has historically been associated with a relatively higher risk of LR due to the extremely distal tumor location and a limited distal margin. Multicenter data evaluating oncological outcomes and risk factors for LR specifically in patients undergoing TaTME combined with ISR remain limited.

Unlike the primary Ta‐Ta‐Mi analysis of the overall TaTME cohort, the present study specifically focused on patients with very low rectal cancer undergoing TaTME combined with ISR. This population represents a technically and oncologically challenging subgroup in whom achieving adequate resection margins while preserving the anal sphincter is particularly important. Therefore, we aimed to evaluate oncological outcomes and identify risk factors for LR specifically in patients undergoing TaTME combined with ISR for lower rectal cancer.

## 2 Materials and Methods

### 2.1 Study Design and Patient Selection

This multicenter retrospective cohort study of combined TaTME and ISR treatment for rectal cancer was conducted at 26 member institutions of the Japan Society of Laparoscopic Colorectal Surgery, a Japanese professional society focused on laparoscopic colorectal resections, between January 2012 and December 2019. No minimum requirement regarding institutional TaTME volume or surgeon experience was specified for participation. Institutional experience was assessed by the annual number of TaTME procedures, and surgeon experience by the cumulative number of TaTME procedures. Exploratory analyses were also performed using cutoffs of 14 cases per year, based on a previous international consensus recommendation [11], and 40 procedures, based on a previously reported learning‐curve study [12]. The inclusion criteria were a histological diagnosis of primary adenocarcinoma or adenosquamous carcinoma, a tumor located within 5 cm of the AV, and clinical stage I–III disease. Patients with non‐primary cancer, multiple cancers, or ulcerative colitis, as well as those with complications from other diseases, or who had undergone surgery for other diseases were excluded. Tumors were classified according to the American Joint Committee on Cancer TNM system [13]. Data were collected using an electronic data capture system.

### 2.2 Procedures

Although TaTME was performed according to a standardized procedure, the choice between a one‐team or two‐team approach and an open, laparoscopic, or robot‐assisted transabdominal approach was left to the discretion of individual institutions. The rectal closure technique was not fully standardized across participating institutions; either a single purse‐string suture or two or more purse‐string sutures, including interrupted sutures, were used according to institutional protocols. Rectal lavage was routinely performed before rectotomy; however, the lavage protocol was not standardized across institutions. The anterior rectourethral muscle was transected after confirming the presence of the prostate on both sides. After circumferential dissection of the internal anal sphincter, TaTME was performed according to the standardized technique described by Arroyave et al. [14] Rectal anastomosis was performed using the handsewn technique. A diverting ileostomy was performed, if necessary.

### 2.3 Follow‐Up

Patients were followed up according to the Japanese Society for Cancer of the Colon and Rectum Guidelines [15]. Follow‐up appointments were scheduled every 3 months for the first 3 years and every 6 months thereafter for 5 years. Each follow‐up included physical examination and measurement of carcinoembryonic antigen and carbohydrate antigen 19‐9. Computed tomography (CT) scans were acquired every 6 months and annually after 3 years for Stage I and II rectal cancers. Total colonoscopy is recommended every 1 or 2 years.

### 2.4 Outcomes

The primary endpoint of the study was the cumulative 3‐year LR rate. The secondary endpoints included the risk factors for LR, LR pattern, positive radial margin (RM) and distal margin (DM) rates, incidence of intraoperative adverse events, incidence of postoperative complications of grade ≥ 3 according to the Clavien–Dindo classification [16], 3‐year recurrence‐free survival (RFS) rate, and 3‐year overall survival (OS) rate. Surgical outcomes, intra‐ and postoperative complications, mortality, reoperation, postoperative hospital stay, number of procedures performed by the surgeon, annual hospital volume, and clinicopathological findings were assessed.

### 2.5 Definitions

LR was defined as pelvic or anastomotic site recurrence on radiographic or endoscopic examination. Distant metastasis was defined as recurrence outside the pelvic cavity. The lateral lymph node area is comprised of the internal iliac and obturator regions. In the original Ta‐Ta‐Mi database, circumferential resection margin (CRM) and RM were not recorded as separate variables but were collected as a single margin‐status variable. Some institutions evaluated the CRM, whereas others assessed the RM according to the Japanese Classification of Colorectal, Appendiceal, and Anal Carcinoma [15]. Therefore, this single margin‐status variable was analyzed as CRM/RM positivity in the present study. CRM/RM positivity was defined as a distance of ≤ 1 mm between the deepest tumor invasion or metastatic lymph node and the adventitial soft tissue margin at the mesenteric resection edge or retroperitoneal surface. A positive DM was defined as a distance of ≤ 1 mm between the distal resection edge and the tumor. Purse‐string suture failure was defined as leakage from the closed rectal cavity during dissection.

### 2.6 Statistical Analysis

Continuous variables were non‐normally distributed and are presented as medians with interquartile ranges (IQRs), whereas categorical variables are expressed as numbers and percentages. The cumulative incidence of LR was estimated using a competing risk model, considering death without recurrence as a competing event, and compared using Gray's test. Patients were stratified according to the presence or absence of risk factors for LR. Missing data were not imputed; patients with missing data for a given variable were excluded from analyses involving that variable.

Cumulative rates of RFS and OS were estimated using the Kaplan–Meier method. The maximum follow‐up duration for survival analysis was 3 years. RFS was defined as the time from surgery to the occurrence of LR and/or distant metastasis or death from any cause, whereas OS was defined as the time from surgery to death from any cause. Univariable analyses were performed to identify potential risk factors for LR. Variables with *p* < 0.05 in the univariate analysis were included in the multivariable cox proportional hazards model to determine independent predictors of LR. As an exploratory analysis, a biologic aggressiveness score was newly constructed to assess the cumulative burden of adverse pathological features associated with LR. Five pathological factors significantly associated with LR in the univariable analysis—histopathological classification, lymphatic invasion, vascular invasion, perineural invasion, and tumor budding—were included in the score. Each factor was assigned one point and summed to generate an unweighted composite score. Equal weighting was used to maintain simplicity and clinical interpretability and to avoid potentially unstable regression‐based weighting given the limited number of LR events. The theoretical score ranged from 0 to 5; however, no patient had all five factors, and the observed scores ranged from 0 to 4. Patients were categorized post hoc into groups with 0, 1, or 2–4 factors because of the small number of patients and LR events in the higher‐score categories. The association between the score and the 3‐year cumulative incidence of LR was then evaluated. A two‐sided *p*‐value < 0.05 was considered statistically significant. All statistical analyses were performed using EZR software [17].

## 3 Results

Overall, 224 patients with lower rectal cancer who underwent TaTME combined with ISR were included in this study. The patient and tumor characteristics are summarized in Table 1. Among them, 161 (71.9%) were male and 87 (38.8%) received neoadjuvant therapy. The median tumor height from the AV was 4.0 (IQR 3.0–5.0) cm. Approximately 44.6% of the tumors were located in the anterior or circumferential wall. Additionally, 14 (6.3%) patients had clinical stage T4 disease, 64 (28.6%) had clinical lymph node metastasis, and 76 (33.9%) received adjuvant therapy.

**TABLE 1:** Patient and tumor characteristics.

Variable	Total cohort
	(*N* = 224)
Age (year), median [IQR]	64 [54–70]
Sex, *n* (%)	
Male	161 (71.9)
Female	63 (28.1)
BMI (kg/m^2^), median [IQR]	23.3 [21.2–25.4]
ASA score, *n* (%)	
I	80 (35.7)
II	133 (59.4)
III	11 (4.9)
IV	0 (0)
Neoadjuvant therapy, *n* (%)	
None	137 (61.2)
NAC	58 (25.9)
RT	1 (0.4)
NACRT	26 (11.6)
TNT	2 (0.9)
Distance from AV (cm), median [IQR]	4 [3–5]
Tumor location	
Anterior	74 (33.0)
Circumferential	26 (11.6)
Not anterior	124 (55.4)
cT^a^, *n* (%)	
1	67 (29.9)
2	57 (25.4)
3	86 (38.4)
4a	7 (3.1)
4b	7 (3.1)
cN^a^, *n* (%)	
0	160 (71.4)
1	48 (21.4)
2	16 (7.1)
cM^a^, *n* (%)	
0	224 (100)
1	0 (0)
cStage^a^, *n* (%)	
I	113 (50.4)
II	47 (21.0)
III	64 (28.6)
IV	0 (0)
CEA, median [IQR]	2.9 [1.8–4.5]
Missing, *n* (%)	1 (0.4)
CA19‐9, median [IQR]	9 [4.9–15.0]
Missing, *n* (%)	2 (0.9)
Adjuvant chemotherapy, *n* (%)	
No	148 (66.1)
Yes	76 (33.9)

Abbreviations: ASA, American Society of Anesthesiologists; AV, anal verge; BMI: body mass index; CA 19‐9, carbohydrate antigen 19‐9; CEA, carcinoembryonic antigen; IQR, interquartile range; NAC, neoadjuvant chemotherapy; NACRT, neoadjuvant chemoradiotherapy; RT, radiotherapy; TNT, total neoadjuvant therapy.

^a^ Tumors were classified according to the American Joint Committee on Cancer TNM system.

The operative and postoperative outcomes of the patients are summarized in Table 2. Lateral pelvic lymph node dissection was performed unilaterally and bilaterally in 19 (8.5%) and 46 (20.5%) patients, respectively. For the abdominal approach, 174 (77.7%) and 48 (21.4%) patients underwent laparoscopic and robotic surgery, respectively. Intraoperative adverse events during transanal dissection included purse‐string failure in six patients (2.7%), rectal perforation in 2 (0.9%), and vaginal injury in two patients (0.9%). Neither of the two patients with rectal perforation developed LR during follow‐up. No urethral and autonomic nerve injuries were observed. Conversion to open surgery was not observed in any case. Clavien–Dindo Grade 2 and 3 postoperative complications occurred in 52 (23.2%) and 21 (9.4%) patients, respectively. No mortality was observed in any case.

**TABLE 2:** Operative and postoperative outcomes.

Variable	Total cohort
	(*N* = 224)
Combined resection, *n* (%)	
Prostate	1 (0.4)
Vagina	0 (0)
Uterus	0 (0)
Other	2 (0.9)
None	221 (98.7)
Lymph node dissection^a^, *n* (%)	
prxD1	0 (0)
prxD2	28 (12.5)
prxD3	196 (87.5)
LLND, *n* (%)	
Unilateral	19 (8.5)
Bilateral	46 (20.5)
No	159 (71.0)
Combined autonomic nerve resection, *n* (%)	
Unilateral	4 (1.8)
Bilateral	5 (2.2)
No	215 (96.0)
Abdominal approach, *n* (%)	
Open	2 (0.9)
Laparoscopic	174 (77.7)
Robotic	48 (21.4)
Diverting stoma, *n* (%)	
Yes	197 (87.9)
No	27 (12.1)
Total operation time (min), median [IQR]	386 [290.5–527.5]
Estimated blood loss (ml), median [IQR]	50 [10–100]
Transfusion, *n* (%)	
Yes	6 (2.7)
No	218 (97.3)
Purse‐string suture^b^, *n* (%)	
Single	44 (19.6)
Double or more	178 (79.5)
Missing	2 (0.9)
Intraoperative adverse events during TaTME, *n* (%)	
Purse‐string failure	6 (2.7)
Visceral injury	
Rectum	2 (0.9)
Vagina	2 (0.9)
Urethra	0 (0)
Autonomic nerve	0 (0)
Bleeding	0 (0)
Conversion	0 (0)
Other	1 (0.4)
Postoperative complications CD Grade 2, *n* (%)	52 (23.2)
Postoperative complications CD Grade 3, *n* (%)	21 (9.4)
Mortality, *n* (%)	0 (0)
Reoperation, *n* (%)	6 (2.7)
Postoperative hospital stay (days), median [IQR]	16 [14–22]
Procedure performed by the surgeon, median [IQR]	12 [5–32]
Annual case numbers of hospital, median [IQR]	12.5 [7–20]

Abbreviations: CD, Clavien–Dindo classification; IQR, interquartile range; LLND, lateral pelvic lymph node dissection; TaTME, transanal total mesorectal excision.

^a^ According to the Japanese Classification of Colorectal, Appendiceal, and Anal Carcinoma.

^b^ The interrupted suture to close the rectum for intersphincteric resection was classified into double or more purse‐string suture.

The pathological findings are summarized in Table 3. The median tumor diameter was 26 (IQR 18–40) mm. Four (1.8%) patients had pathological T4 stage disease, and 45 (20.1%) were positive for pathological lymph node metastasis. Lateral pelvic lymph node metastasis was observed in 7 (3.1%). Perineural invasion was observed in 30 (13.4%) patients, and CRM/RM was positive in 8 (3.6%) patients. DM was positive in 2 (0.9%). A good pathological response (moderate to marked effect [15]) was observed in 32 (36.8%) of the 87 patients who received neoadjuvant therapy.

**TABLE 3:** Pathological outcomes.

Variable	Total cohort
	(*N* = 224)
Histological type, *n* (%)	
Differentiated	212 (94.6)
Undifferentiated	7 (3.1)
Other	5 (2.2)
Tumor diameter (mm), median [IQR]	26 [18–40]
ypT^a^, *n* (%)	
0/is	19 (8.5)
1	69 (30.8)
2	72 (32.1)
3	60 (26.8)
4a	3 (1.3)
4b	1 (0.4)
ypN^a^, *n* (%)	
0	179 (79.9)
1	38 (17.0)
2	7 (3.1)
ypM^a^, *n* (%)	
0	224 (100)
1	0 (0)
ypStage^a^, *n* (%)	
0	21 (9.4)
I	123 (54.9)
II	35 (15.6)
III	45 (20.1)
IV	0 (0)
Positive LLN metastasis, *n* (%)	7 (3.1)
Lymphatic invasion, *n* (%)	
Absent	148 (66.1)
Present	76 (33.9)
Vascular invasion, *n* (%)	
Absent	113 (50.4)
Present	111 (49.6)
Budding^b^, *n* (%)	
BD1	94 (42.0)
BD2	14 (6.3)
BD3	6 (2.7)
BDX	110 (49.1)
Perineural invasion, *n* (%)	
Absent	145 (64.7)
Present	30 (13.4)
unknown	49 (21.9)
CRM/RM, *n* (%)	
Negative (> 1 mm)	202 (90.2)
Positive (≤ 1 mm)	8 (3.6)
unknown	14 (6.3)
DM, *n* (%)	
Negative (> 1 mm)	222 (99.1)
Positive (≤ 1 mm)	2 (0.9)
Number of LNs harvested, median [IQR]	17 [11–26]
Pathological response^b^, *n* (%)	
Good	32 (14.3)
Poor	54 (24.1)

Abbreviations: CRM/RM, circumferential resection margin or radial margin; DM, distal margin; IQR, interquartile range; LLN, lateral pelvic lymph node; LNs, lymph nodes; LR, local recurrence; Ly, lymphatic invasion; OS, overall survival; V, vascular invasion.

^a^ Tumors were classified according to the American Joint Committee on Cancer TNM system.

^b^ According to the Japanese Classification of Colorectal, Appendiceal, and Anal Carcinoma.

The oncological outcomes of patients are summarized in Table 4. After a median follow‐up of 4.3 (IQR, 3.5–5.0) years, LR was identified in 16 patients within 3 years. The LR locations were the central (*n* = 5, 2.3%), lateral pelvis (*n* = 9, 4.2%), and anastomotic sites (*n* = 2, 0.9%). A multifocal pattern was observed in 2 (12.5%) of the 16 patients with LR. The median time to LR was 1.9 (IQR 1.0–2.6) years. The 3‐year cumulative LR rates were 7.3% (95% confidence interval [CI] 4.5–11.7).

**TABLE 4:** Oncological outcomes.

Variable	Total cohort
	(*N* = 224)
Median follow‐up duration, years [IQR]	4.3 [3.5–5.0]
3‐year LR, % (95% CI)	7.3 (4.5–11.7)
Central pelvis	2.3 (1.0–5.5)
Lateral pelvis	4.2 (2.2–8.0)
Anastomosis	0.9 (0.2–3.6)
Multifocal	1.0 (0.2–3.8)
Pattern of 3‐year LR, *n* (%)	
Unifocal	14/16 (87.5)
Multifocal	2/16 (12.5)
Time to LR (years), median [IQR]	1.9 [1.0–2.6]
3‐year RFS, % (95% CI)	79.5 (73.5–84.3)
3‐year OS, % (95% CI)	98.1 (95.1–99.3)

Abbreviations: CI, confidence interval; IQR, interquartile range; LR, local recurrence; OS, overall survival; RFS, recurrence‐free survival.

A treatment diagram of patients with LR is shown in Figure 1. Sixteen patients had LR; among them, one was diagnosed with systemic disease progression. Among the remaining 15 patients who underwent LR only, eight underwent salvage surgery, all of whom were alive and disease‐free at the end of the follow‐up period. Five patients received palliative chemotherapy, and one received radiotherapy for LR. One patient did not receive treatment. Four of the eight patients died during follow‐up, whereas four were alive with the disease at the time of data extraction. One patient experienced distant recurrence during LR diagnosis, underwent chemotherapy, and died during follow‐up.

**FIGURE 1:**
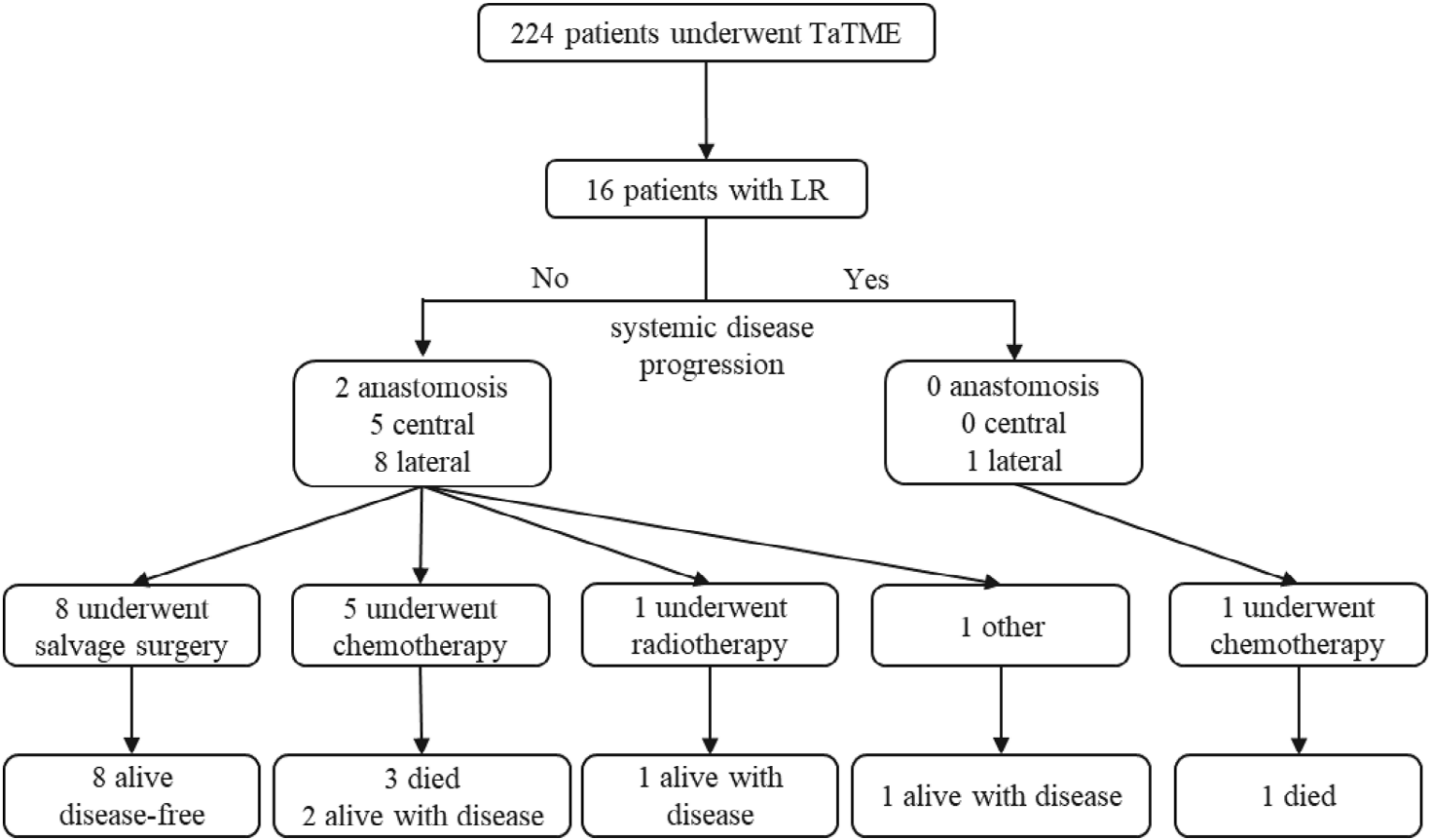
Flow diagram illustrating treatment pathways and oncological outcomes in patients with local recurrence (LR).

The Kaplan–Meier survival curves and cumulative incidence functions with Gray's test are shown in Figure 2. The cumulative LR proportion was 7.3% (95% CI 4.5–11.7) at 3 years for the total cohort (Figure 2A). The 3‐year RFS and OS were 79.5% (95% CI 73.5–84.3) and 98.1% (95% CI 95.1–99.3), respectively (Figure 2B,C).

**FIGURE 2:**
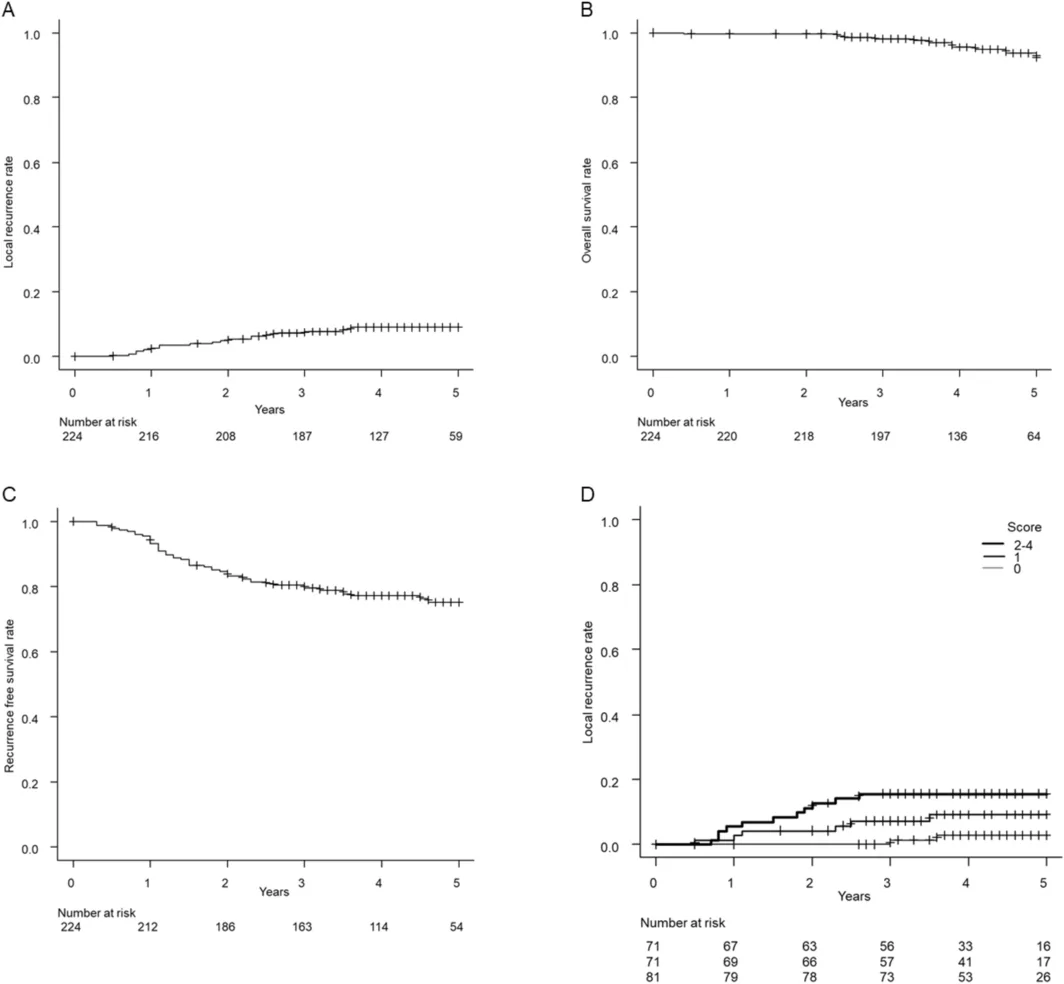
Kaplan–Meier survival curves showing (A) cumulative local recurrence (LR) rate, (B) recurrence‐free survival (RFS) rate, (C) overall survival (OS) rate, and (D) cumulative LR rates stratified based on biologic aggressiveness scores.

Univariate and multivariate analyses of potential risk factors for LR are presented in Table 5. Six potential risk factors, including histopathological classification, lymphatic invasion, vascular invasion, perineural invasion, budding, and CRM/RM, associated with LR were identified in the univariate analysis. No significant differences were found in the univariate analysis of T, N, stage, the number of TaTMEs performed by the surgeon, hospital TaTME volume, or the use of preoperative or postoperative treatment. The median surgeon experience was 12 TaTME procedures, and the median annual institutional TaTME volume was 12.5 cases. No significant differences in LR, RFS, or OS were observed using cutoffs of 40 procedures for surgeon experience or 14 cases per year for institutional volume (Table S1). The multivariate regression model included the histopathological classification, lymphatic invasion, vascular invasion, perineural invasion, budding, and CRM/RM. The final multivariate regression analysis identified CRM/RM as the only significant factor associated with LR, with a hazard ratio of 8.59 (95% confidence interval: 1.21–61.15). The 3‐year LR rate was 6.6% in patients without CRM/RM positivity and 37.5% in those with CRM/RM positivity (Figure S1). The cumulative LR curves according to the biologic aggressiveness score are shown in Figure 2D. The cumulative 3‐year LR rate was 1.4% in patients with no adverse factors, 7.3% in those with one factor, and 15.6% in those with two–four factors. A higher LR rate was observed in patients with higher biologic aggressiveness scores. Cox regression analyses for RFS and OS are presented in the Tables S2 and S3, respectively. In multivariable analysis, pathological stage, tumor budding, and CRM/RM positivity were associated with worse RFS.

**TABLE 5:** Univariable and multivariable analyses of potential risk factors for LR.

Variable	Univariate	*p*	Multivariate	*p*
	Hazard ratio (95% CI)		Hazard ratio (95% CI)	
Age	0.98 (0.94–1.02)	0.31		
Sex				
Male	Reference			
Female	1.18 (0.39–3.67)	0.76		
BMI	1.00 (0.85–1.17)	0.97		
Neoadjuvant therapy				
No	Reference			
Yes	0.35 (0.10–1.23)	0.1		
Distance from AV	0.74 (0.50–1.08)	0.12		
Tumor location				
Anterior/Circumferential	Reference			
Not anterior	0.95 (0.36–2.54)	0.92		
cT^a^				
0–3	Reference			
4a,4b	0.96 (0.13–7.08)	0.97		
cN^a^				
0	Reference			
1,2	1.17 (0.41–3.36)	0.77		
cStage^a^				
0–I	Reference			
II–III	0.60 (0.22–1.64)	0.32		
ypT^a^				
0–3	Reference			
4a,4b	4.28 (0.50–36.37)	0.18		
ypN^a^				
0	Reference			
1,2	2.39 (0.88–6.49)	0.09		
ypStage^a^				
0–I	Reference			
II–IV	2.38 (0.89–6.36)	0.09		
CEA	1.00 (0.96–1.05)	0.88		
CA19‐9	1.00 (0.98–1.02)	0.79		
Adjuvant chemotherapy				
No	Reference			
Yes	1.91 (0.72–5.04)	0.19		
Purse‐string suture				
Single	Reference			
Double or more	1.85 (0.41–8.28)	0.42		
Intraoperative TaTME adverse events				
No	Reference			
Yes	0 (0)	0		
Purse‐string failure				
No	Reference			
Yes	0 (0)	0		
Visceral injury				
No	Reference			
Yes	0 (0)	0		
Tumor diameter	0.99 (0.97–1.01)	0.47		
Histopathological type				
Differentiated	Reference			
Undifferentiated	4.75 (1.14–19.8)	0.03	2.87 (0.48–17.12)	0.25
Lymphatic invasion				
Absent	Reference			
Present	4.40 (1.54–12.57)	0.006	1.95 (0.34–11.34)	0.46
Vascular invasion				
Absent	Reference			
Present	3.11 (1.01–9.54)	0.050	2.93 (0.26–33.12)	0.38
Perineural invasion				
Negative	Reference			
Positive	4.37 (1.49–12.8)	0.007	1.32 (0.18–9.67)	0.79
Budding^b^				
BD1	Reference			
BD2‐3	6.83 (1.87–24.9)	0.004	2.04 (0.42–9.81)	0.37
CRM/RM				
Negative	Reference			
Positive	7.50 (2.02–27.91)	0.003	8.59 (1.21–61.15)	0.03
DM				
Negative	Reference			
Positive	12.57 (1.04–152)	0.051	—	—
Number of retrieved lymph nodes	0.96 (0.91–1.02)	0.19		
Number of procedures performed by the surgeon	1.00 (0.98–1.02)	0.91		
Annual case numbers of hospital	1.00 (0.96–1.05)	0.84		

Abbreviations: AV, anal verge; BMI, body mass index; CA 19‐9, carbohydrate antigen 19‐9; CEA, carcinoembryonic antigen; CI, confidence interval; CRM/RM, circumferential resection margin or radial margin; DM, distal margin; LR, local recurrence; Ly, lymphatic invasion; TaTME, transanal total mesorectal excision; V, vascular invasion.

^a^ Tumors were classified according to the American Joint Committee on Cancer TNM system.

^b^ According to the Japanese Classification of Colorectal, Appendiceal, and Anal Carcinoma.

## 4 Discussion

This multicenter observational cohort study evaluated local LR rates in 224 patients who underwent TaTME combined with ISR for lower rectal cancer across 26 institutions participating in Japan Society for Laparoscopic Colorectal Surgery. The present analysis extends the primary Ta‐Ta‐Mi study by focusing on a more narrowly defined subgroup of patients with very low rectal cancer who underwent ISR with hand‐sewn anastomosis. This population is technically challenging because adequate margins must be achieved while preserving the anal sphincter. Our findings therefore provide additional information on local control and factors associated with LR after TaTME combined with ISR. To our knowledge, this represents the largest nationwide dataset specifically examining the oncological outcomes of TaTME combined with ISR in Japan. The present study demonstrated acceptable oncological outcomes, comparable to those reported in recent TaTME series. These findings suggest that the combination of TaTME and ISR may achieve acceptable oncological outcomes in selected patients with lower rectal cancer.

Among the 16 patients with LR, two patients had multifocal recurrence patterns. Salvage surgery was feasible in a subset of patients with isolated local recurrence, suggesting that selected patients may benefit from potentially curative salvage treatment. These findings contrast with those of the Norwegian national audit, which reported an estimated LR rate of 11.6% after 2.4 years, with multifocal or extensive recurrence patterns observed in two‐thirds of patients [4]. Our findings are more consistent with previous studies using purse‐string closure and rectal lavage, supporting the notion that TaTME can achieve accurate distal dissection and adequate margin clearance, particularly in tumors of the lower rectum [1, 2, 8, 9, 10].

LR remains a key metric for evaluating oncological outcomes after rectal cancer surgery. In the present study, the overall 3‐year LR rate was 7.3%. A systematic review and meta‐analysis of 15 studies reported median LR rates of 7.79% after ISR and 15.71% after APR [18]. Although recent studies have reported lower LR rates, including a 5‐year LR rate of 5.1% after robotic ISR [19] and a 3‐year LR rate of 3.6% in randomized trial of TaTME for low anterior resection [20], direct comparison is difficult. In the present study, lateral pelvic recurrence, including recurrence in the internal iliac and obturator regions, was classified as local recurrence. Such lateral pelvic recurrence represents a distinct recurrence pattern that may reflect anatomical factors beyond the technical quality of the TaTME procedure itself.

Multivariable analysis identified CRM/RM positivity as the key risk factor for LR. In the present study, the LR rates differed significantly according to CRM/RM status, highlighting the importance of achieving clear margins even when the enhanced visualization provided by TaTME is used. In contrast, pathological factors, including histopathological classification, lymphatic invasion, vascular invasion, perineural invasion, and tumor budding, are unlikely to be directly influenced by surgical performance and instead reflect the intrinsic biological aggressiveness of the tumor. To explore the cumulative impact of these biological factors, we constructed a biological aggressiveness score. A higher biological aggressiveness score was associated with a stepwise increase in the cumulative incidence of LR. These findings suggest that both surgical factors, represented by CRM/RM status, and tumor biological characteristics may contribute to LR after TaTME with ISR. However, because this score was constructed post hoc using factors identified in the same cohort and was not externally validated, it should be regarded as an exploratory measure rather than a validated predictive model. Further validation in independent cohorts is required before clinical application.

Several studies have investigated risk factors for LR after TaTME. Reported predictors include body mass index (BMI), CRM/RM, purse‐string failure, neoadjuvant therapy, pathological stage, and surgical experience at both the surgeon and institutional levels [11, 12, 21, 22, 23]. Roodbeen et al. [5] reported that male sex, threatened RM on baseline magnetic resonance imaging (MRI), pathological stage III disease, and positive CRM were independent risk factors for LR. Although a threatened RM on baseline MRI is an important predictor of LR in rectal cancer surgery, this variable was not available in the present dataset. Intraoperative purse‐string failure was associated with LR in our cohort; however, no significant difference was observed due to the limited number of cases. Neither annual institutional TaTME volume nor surgeon experience was significantly associated with LR, RFS, or OS. However, because no validated threshold for institutional or surgeon experience has been established for oncological outcomes, the present study cannot define a minimum volume or experience requirement for TaTME with ISR.

The current findings demonstrated favorable oncological outcomes after TaTME combined with ISR, with 3‐year RFS and OS rates of 79.5% and 98.1%, respectively. Liu et al. [24] reported 3‐year RFS and OS rates of 86.3% and 96.7% following TaTME combined with ISR, while Xu et al. [9] demonstrated improved disease‐free survival (DFS) after TaTME combined with ISR compared with laparoscopic ISR in patients with ultralow rectal cancer. In addition, systematic reviews have reported 5‐year OS and DFS rates of approximately 80%–86% and 72%–79%, respectively, after ISR [25]. In the present cohort, survival outcomes were comparable to those reported in previous TaTME series, despite a relatively high proportion of locally advanced tumors. Advances in multidisciplinary treatment, including neoadjuvant chemoradiotherapy and total neoadjuvant therapy, have expanded the indications for sphincter‐preserving surgery in lower rectal cancer [26, 27]. TaTME may further facilitate this procedure by improving access and visualization in the deep pelvis. Although TaTME combined with ISR remains technically demanding and carries a potential risk of urethral injury [28], no urethral injuries were observed in the present study. This finding further supports the technical safety of this approach. The postoperative complication rate was also acceptable, suggesting that this approach may be feasible in selected patients.

Although anorectal functional outcomes after TaTME combined with ISR were not evaluated in the present study, previous reports have provided important insights into postoperative bowel function. Liu et al. [24] reported that low anterior resection syndrome (LARS) occurred in 45.3% of patients undergoing TaTME combined with ISR, while acceptable anal function was preserved in 70.9% of cases. Furthermore, a propensity score‐matched study comparing ISR with very low anterior resection demonstrated that ISR was associated with a higher incidence of fecal incontinence and anal pain but did not significantly increase the prevalence of LARS, fecal urgency, or clustering [29]. In addition, a recent meta‐analysis comparing laparoscopic TME and TaTME reported comparable postoperative anorectal functional outcomes and quality of life (QOL) between the two approaches [30]. Taken together, these findings suggest that, although sphincter‐preserving procedures such as TaTME combined with ISR may increase certain functional symptoms, overall bowel function and QOL may remain acceptable in carefully selected patients. Nevertheless, the absence of functional assessment in the present study represents an important limitation, and further prospective studies incorporating standardized functional and QOL evaluations are warranted.

Some study limitations warrant mention. First, the retrospective design may have introduced selection bias. Second, although a standardized protocol was referenced, some surgical procedures, including the abdominal approach (laparoscopic, robotic, or open), rectal closure technique, and lavage protocol, were not strictly standardized across participating institutions, which may have influenced the oncological outcomes. Third, pathological evaluations were not centrally reviewed, and margin assessment was not standardized across participating institutions. In the original database, CRM and RM were collected as a single margin‐status variable and could not be distinguished at the individual patient level. Therefore, separate analyses of CRM and RM positivity, including sensitivity analyses, could not be performed. Given the anatomical and clinical differences between CRM and RM, this limitation should be considered when interpreting the association between CRM/RM positivity and LR. Fourth, some clinicopathological variables had missing data, including PN and CRM/RM status, which were unavailable in 49 and 14 patients, respectively. Preoperative CEA and CA19‐9 levels were also missing in one and two patients, respectively. Because these missing data were not available in the original database, potential bias due to missing data cannot be excluded. Fifth, baseline MRI findings regarding threatened circumferential resection margins were not consistently available, and preoperative MRI‐EMVI status was not collected in the original database. Therefore, the association between MRI‐EMVI and LR could not be evaluated, limiting a comprehensive assessment of preoperative oncological risk. Finally, postoperative functional outcomes, including anorectal function and LARS, and QOL were not assessed. In addition, longer follow‐up is required to evaluate long‐term oncological outcomes, including distant metastasis. Despite these limitations, this nationwide multicenter cohort study with a relatively large sample size provides important insights into oncological outcomes and risk factors for LR after TaTME combined with ISR, highlighting the importance of careful patient selection, surgical precision, and comprehensive postoperative management.

In conclusion, TaTME combined with ISR appears to be a feasible sphincter‐preserving option for patients with lower rectal cancer, with acceptable LR rates and favorable RFS and OS outcomes. However, achieving margin‐negative resection and providing careful postoperative surveillance in patients with adverse pathological features are essential for optimizing oncological outcomes. Future prospective studies are warranted to validate these findings and further refine risk stratification.

## Author Contributions

**Kan Tanabe:** formal analysis, writing – original draft, writing – review and editing, investigation. **Shinichiro Mori:** conceptualization, writing – original draft, writing – review and editing, investigation. **Kenji Baba:** investigation, resources, writing – review and editing. **Takeru Matsuda:** conceptualization, investigation, writing – review and editing. **Ichiro Takemasa:** conceptualization, supervision, writing – review and editing. **Koji Okabayashi:** investigation, resources, writing – review and editing. **Chikayoshi Tani:** investigation, writing – review and editing, resources. **Tatsuya Manabe:** investigation, resources, writing – review and editing. **Yoshihiro Kakeji:** conceptualization, resources, writing – review and editing. **Takao Ohtsuka:** resources, supervision, writing – review and editing. **Seiichiro Yamamoto:** conceptualization, supervision, writing – review and editing. **Takeshi Naitoh:** conceptualization, supervision, writing – review and editing.

## Funding

This work was supported by a grant from the Japanese Foundation for the Research and Promotion of Endoscopy. The funding organization was not involved in any stage of the design or conduct of the study; collection, management, analysis, and interpretation of the data; or final approval of the article.

## Ethics Statement

This study was approved by the Institutional Review Board and Ethics Committee of Kobe University Graduate School of Medicine (IRB reference code: B220092). The need for informed consent was waived due to the anonymity of the study data. However, in accordance with Japanese ethical regulations, the Japanese Personal Information Protection Law, and instructions from the ethics committees of each institution, consent was obtained via the opt‐out route. Information regarding the purpose and methodology of the study was disclosed on the hospital noticeboard and website, and opportunities to opt out were provided as much as possible. The study was conducted in accordance with the Declaration of Helsinki.

## Conflicts of Interest

The authors declare no conflicts of interest.

## Supporting information


**Figure S1:** Cumulative incidence of local recurrence (LR) by circumferential margin (CRM)/radial margin (RM) status.

**Table S1:** Oncological outcomes according to surgeon experience and annual hospital volume.

**Table S2:** Univariable and multivariable analyses of risk factors for RFS.

**Table S3:** Univariable and multivariable analyses of risk factors for OS.

## Data Availability

The data that support the findings of this study are available from Kobe University Graduate School of Medicine, but restrictions apply to the availability of these data, which were used under the license for the current study, and so are not publicly available. However, data are available from T. Matsuda upon reasonable request and with permission from the Kobe University Graduate School of Medicine.
