# Supplementary material for: Local Recurrence and Oncological Outcomes After Transanal Total Mesorectal Excision With Intersphincteric Resection for Lower Rectal Cancer: A Nationwide Multicenter Cohort Study (Ta‐Ta‐Mi Study)
Source: Ann Gastroenterol Surg. 2026 Oct 31:10.1002/ags3.70304. Online ahead of print. doi: 10.1002/ags3.70304 (PMC13722975; doi:10.1002/ags3.70304)
Supplement: Supplementary file 1 [file AGS3-9999-0-s004.docx]

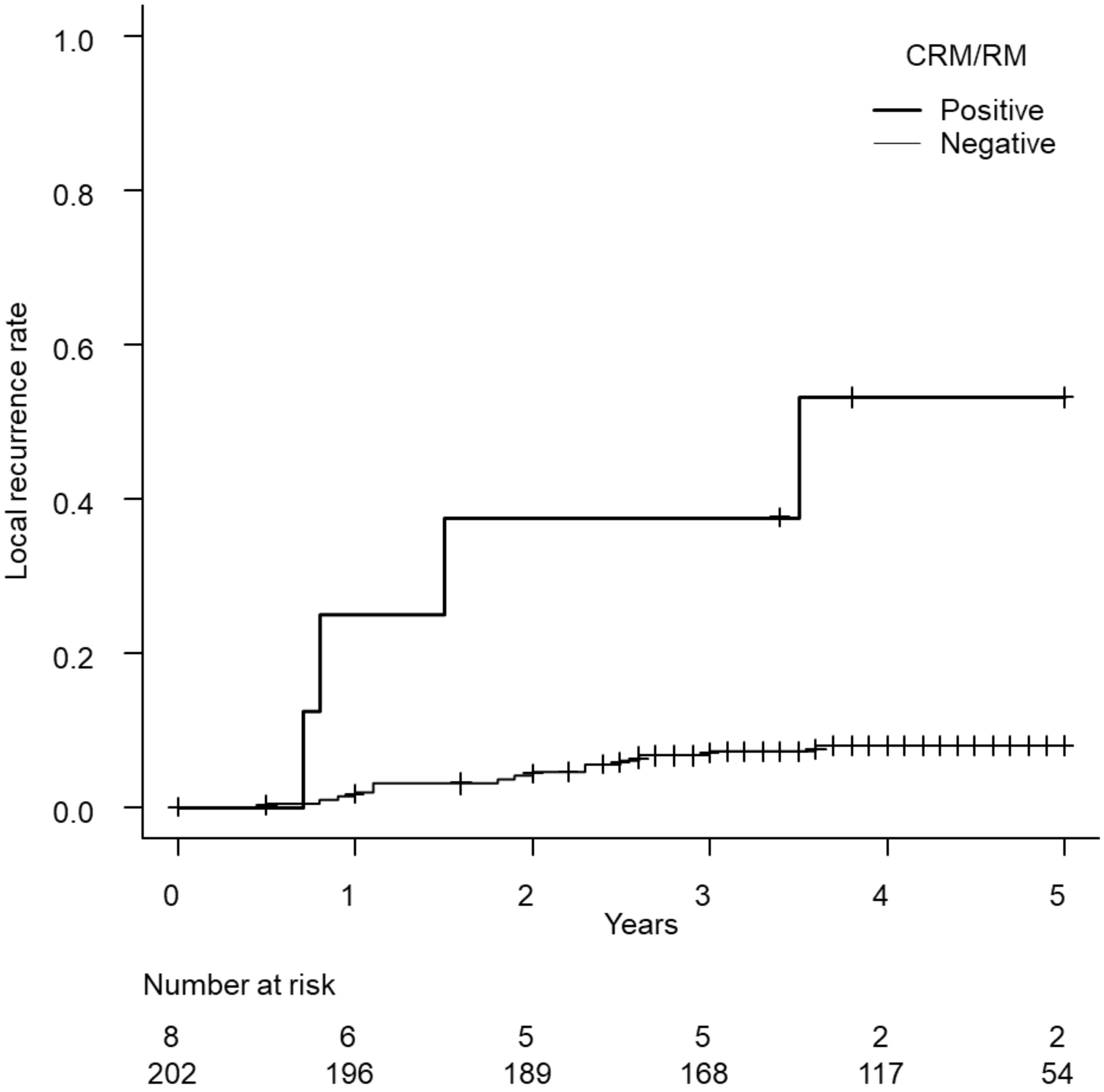

CRM/RM
Positive
Negative
Local recurrence rate
1.0
0.8
0.6
0.4
0.2
0.0
0
1
2
3
4
5
Years
Number at risk
8 6 5 5 2 2
202 196 189 168 117 54
