# Supplementary material for: Local Recurrence and Oncological Outcomes After Transanal Total Mesorectal Excision With Intersphincteric Resection for Lower Rectal Cancer: A Nationwide Multicenter Cohort Study (Ta‐Ta‐Mi Study)
Source: Ann Gastroenterol Surg. 2026 Oct 31:10.1002/ags3.70304. Online ahead of print. doi: 10.1002/ags3.70304 (PMC13722975; doi:10.1002/ags3.70304)
Supplement: Supplementary file 1 [file AGS3-9999-0-s001.docx]

Supplemental Table 3. Univariable and multivariable analyses of potential risk factors for OS

|  | Univariate |  | Multivariate |  |
| --- | --- | --- | --- | --- |
| Variable | Hazard ratio (95% CI) | p-value | Hazard ratio (95% CI) | p-value |
| Age | 0.94 (0.87–1.02) | 0.14 |  |  |
| Sex |  |  |  |  |
| Male | Reference |  |  |  |
| Female | 0 (0) | 0 |  |  |
| BMI | 1.10 (0.84–1.45) | 0.48 |  |  |
| Neoadjuvant therapy |  |  |  |  |
| No | Reference |  |  |  |
| Yes | 0.50 (0.05–4.85) | 0.55 |  |  |
| Distance from AV | 0.78 (0.34–1.77) | 0.55 |  |  |
| Tumor location |  |  |  |  |
| Anterior/circumferential | Reference |  |  |  |
| Not anterior | 1.25 (0.18–8.90) | 0.82 |  |  |
| cT* |  |  |  |  |
| 0–3 | Reference |  |  |  |
| 4a,4b | 0 (0) | 0 |  |  |
| cN* |  |  |  |  |
| 0 | Reference |  |  |  |
| 1,2 | 0.82 (0.09–7.92) | 0.87 |  |  |
| cStage* |  |  |  |  |
| 0-I | Reference |  |  |  |
| II-III | 0.99 (0.14–7.05) | 0.99 |  |  |
| ypT* |  |  |  |  |
| 0–3 | Reference |  |  |  |
| 4a,4b | 17.29 (1.80–166.3) | 0.01 | 8.39 (0.36-194.5) | 0.18 |
| ypN* |  |  |  |  |
| 0 | Reference |  |  |  |
| 1,2 | 1.31 (0.14–12.57) | 0.82 |  |  |
| ypStage* |  |  |  |  |
| 0-I | Reference |  |  |  |
| II-IV | 0.60 (0.06–5.75) | 0.66 |  |  |
| CEA | 1.11 (0.97–1.27) | 0.12 |  |  |
| CA19-9 | 0.94 (0.81–1.10) | 0.45 |  |  |
| Adjuvant chemotherapy |  |  |  |  |
| No | Reference |  |  |  |
| Yes | 0.61 (0.06–5.90) | 0.67 |  |  |
| Purse-string suture |  |  |  |  |
| Single | Reference |  |  |  |
| Double or more | 0.26 (0.04–1.86) | 0.18 |  |  |
| Intraoperative  TaTME adverse events |  |  |  |  |
| No | Reference |  |  |  |
| Yes | 0 (0) | 0 |  |  |
| Purse-string failure |  |  |  |  |
| No | Reference |  |  |  |
| Yes | 0 (0) | 0 |  |  |
| Visceral injury |  |  |  |  |
| No | Reference |  |  |  |
| Yes | 0 (0) | 0 |  |  |
| Tumor diameter | 1.01 (0.96–1.06) | 0.67 |  |  |
| Histopathological classification |  |  |  |  |
| Tub1, tub2, pap, other | Reference |  |  |  |
| Por, muc, sig | 9.74 (1.01–93.7) | 0.049 | 3.36 (0.14-77.79) | 0.45 |
| Lymphatic invasion |  |  |  |  |
| Absent | Reference |  |  |  |
| Present | 1.40 (0.17–11.76) | 0.758 |  |  |
| Vascular invasion |  |  |  |  |
| Absent | Reference |  |  |  |
| Present | 3.05 (0.32–29.30) | 0.33 |  |  |
| Perineural invasion |  |  |  |  |
| Negative | Reference |  |  |  |
| Positive | 1.66 (0.17–15.99) | 0.66 |  |  |
| Budding** |  |  |  |  |
| BD1 | Reference |  |  |  |
| BD2-3 | 0 (0) | 0 |  |  |
| CRM/RM |  |  |  |  |
| Negative | Reference |  |  |  |
| Positive | 7.90 (0.82–75.93) | 0.07 |  |  |
| DM |  |  |  |  |
| Negative | Reference |  |  |  |
| Positive | 0 (0) | 0 |  |  |
| Number of retrieved lymph nodes | 0.94 (0.84–1.05) | 0.30 |  |  |
| Number of procedures performed by the surgeon | 1.01 (0.99–1.04) | 0.27 |  |  |
| Annual case numbers of hospital | 1.03 (0.89–1.19) | 0.71 |  |  |
| *Tumors were classified according to the American Joint Committee on Cancer TNM system. **According to the Japanese Classification of Colorectal, Appendiceal, and Anal Carcinoma. AV: anal verge; BMI: body mass index; CA 19-9: carbohydrate antigen 19-9; CEA: carcinoembryonic antigen; CI: confidence interval; CRM/RM: circumferential resection margin or radial margin; DM: distal margin; LR: local recurrence; Ly: lymphatic invasion; TaTME: transanal total mesorectal excision; V: vascular invasion | | | | |
