# Supplementary material for: Local Recurrence and Oncological Outcomes After Transanal Total Mesorectal Excision With Intersphincteric Resection for Lower Rectal Cancer: A Nationwide Multicenter Cohort Study (Ta‐Ta‐Mi Study)
Source: Ann Gastroenterol Surg. 2026 Oct 31:10.1002/ags3.70304. Online ahead of print. doi: 10.1002/ags3.70304 (PMC13722975; doi:10.1002/ags3.70304)
Supplement: Supplementary file 2 [file AGS3-9999-0-s002.docx]

Supplemental Table 1. Oncological outcomes according to surgeon experience and annual hospital volume

| Surgeon experience | | | |
| --- | --- | --- | --- |
| Outcome | <40 procedures | ≥40 procedures | p-value |
| Patients, n (%) | 192 (85.7) | 32 (14.3) | - |
| 3-year LR, % (95% CI) | 7.0 (4.1–11.8) | 6.3 (1.6–22.7) | 0.89 |
| 3-year RFS, % (95% CI) | 80.8 (74.4–85.7) | 81.2 (62.9–91.1) | 0.97 |
| 3-year OS, % (95% CI) | 98.4 (95.0–99.5) | 96.7 (78.6–99.5) | 0.57 |
| Annual hospital volume | | | |
| Outcome | <14 cases | ≥14 cases | p-value |
| Patients, n (%) | 121 (54.0) | 103 (46.0) | - |
| 3-year LR, % (95% CI) | 7.7 (4.1–14.2) | 6.8 (3.4–14.0) | 0.82 |
| 3-year RFS, % (95% CI) | 78.1 (69.5–84.5) | 83.3 (74.5–89.3) | 0.35 |
| 3-year OS, % (95% CI) | 98.3 (93.4–99.6) | 97.9 (91.9–99.5) | 0.87 |
| LR: local recurrence; OS: overall survival; RFS: recurrence-free survival | | | |
