# Supplementary material for: Local Recurrence and Oncological Outcomes After Transanal Total Mesorectal Excision With Intersphincteric Resection for Lower Rectal Cancer: A Nationwide Multicenter Cohort Study (Ta‐Ta‐Mi Study)
Source: Ann Gastroenterol Surg. 2026 Oct 31:10.1002/ags3.70304. Online ahead of print. doi: 10.1002/ags3.70304 (PMC13722975; doi:10.1002/ags3.70304)
Supplement: Supplementary file 3 [file AGS3-9999-0-s003.docx]

Supplemental Table 2. Univariable and multivariable analyses of potential risk factors for RFS

|  | Univariate |  | Multivariate |  |
| --- | --- | --- | --- | --- |
| Variable | Hazard ratio (95% CI) | p-value | Hazard ratio (95% CI) | p-value |
| Age | 0.99 (0.96–1.02) | 0.41 |  |  |
| Sex |  |  |  |  |
| Male | Reference |  |  |  |
| Female | 1.33 (0.65–2.69) | 0.43 |  |  |
| BMI | 1.06 (0.97–1.15) | 0.19 |  |  |
| Neoadjuvant therapy |  |  |  |  |
| No | Reference |  |  |  |
| Yes | 1.09 (0.83–1.44) | 0.53 |  |  |
| Distance from AV | 0.86 (0.66–1.12) | 0.27 |  |  |
| Tumor location |  |  |  |  |
| Anterior/circumferential | Reference |  |  |  |
| Not anterior | 0.70 (0.37–1.31) | 0.27 |  |  |
| cT* |  |  |  |  |
| 0–3 | Reference |  |  |  |
| 4a,4b | 0.32 (0.04–2.34) | 0.26 |  |  |
| cN* |  |  |  |  |
| 0 | Reference |  |  |  |
| 1,2 | 1.76 (0.95–3.24) | 0.07 |  |  |
| cStage* |  |  |  |  |
| 0-I | Reference |  |  |  |
| II-III | 1.63 (0.89–3.01) | 0.12 |  |  |
| ypT* |  |  |  |  |
| 0–3 | Reference |  |  |  |
| 4a,4b | 1.47 (0.20–10.65) | 0.71 |  |  |
| ypN* |  |  |  |  |
| 0 | Reference |  |  |  |
| 1,2 | 2.85 (1.55–5.26) | 0.0007 | 0.23 (0.06-0.89) | 0.03 |
| ypStage* |  |  |  |  |
| 0-I | Reference |  |  |  |
| II-IV | 3.41 (1.83–6.32) | 0.0001 | 6.70 (1.53-29.38) | 0.01 |
| CEA | 1.02 (0.99–1.06) | 0.13 |  |  |
| CA19-9 | 1.02 (1.00–1.03) | 0.02 | 1.03 (0.96-1.10) | 0.40 |
| Adjuvant chemotherapy |  |  |  |  |
| No | Reference |  |  |  |
| Yes | 1.89 (1.04–3.43) | 0.04 | 0.74 (0.20-2.68) | 0.64 |
| Purse-string suture |  |  |  |  |
| Single | Reference |  |  |  |
| Double or more | 0.82 (0.41–1.67) | 0.59 |  |  |
| Intraoperative  TaTME adverse events |  |  |  |  |
| No | Reference |  |  |  |
| Yes | 0 (0) | 0 |  |  |
| Purse-string failure |  |  |  |  |
| No | Reference |  |  |  |
| Yes | 0 (0) | 0 |  |  |
| Visceral injury |  |  |  |  |
| No | Reference |  |  |  |
| Yes | 0 (0) | 0 |  |  |
| Tumor diameter | 1.01 (1.00–1.03) | 0.12 |  |  |
| Histopathological classification |  |  |  |  |
| Tub1, tub2, pap, other | Reference |  |  |  |
| Por, muc, sig | 2.57 (0.79–8.3) | 0.12 |  |  |
| Lymphatic invasion |  |  |  |  |
| Absent | Reference |  |  |  |
| Present | 2.61 (1.43–4.76) | 0.002 | 1.69 (0.43-6.61) | 0.45 |
| Vascular invasion |  |  |  |  |
| Absent | Reference |  |  |  |
| Present | 2.23 (1.18–4.22) | 0.01 | 1.52 (0.41-5.60) | 0.53 |
| Perineural invasion |  |  |  |  |
| Negative | Reference |  |  |  |
| Positive | 2.83 (1.41–5.70) | 0.004 | 0.78 (0.23-2.64) | 0.70 |
| Budding** |  |  |  |  |
| BD1 | Reference |  |  |  |
| BD2-3 | 5.35 (2.26–12.6) | 0.0001 | 4.35 (1.17-16.10) | 0.03 |
| CRM/RM |  |  |  |  |
| Negative | Reference |  |  |  |
| Positive | 4.89 (1.92–12.48) | 0.0009 | 9.34 (2.07-42.17) | 0.004 |
| DM |  |  |  |  |
| Negative | Reference |  |  |  |
| Positive | 4.12 (0.57–30.0) | 0.16 |  |  |
| Number of retrieved lymph nodes | 0.99 (0.97–1.02) | 0.62 |  |  |
| Number of procedures performed by the surgeon | 1.00 (0.99–1.01) | 0.46 |  |  |
| Annual case numbers of hospital | 1.00 (0.97–1.03) | 0.96 |  |  |
| *Tumors were classified according to the American Joint Committee on Cancer TNM system. **According to the Japanese Classification of Colorectal, Appendiceal, and Anal Carcinoma. AV: anal verge; BMI: body mass index; CA 19-9: carbohydrate antigen 19-9; CEA: carcinoembryonic antigen; CI: confidence interval; CRM/RM: circumferential resection margin or radial margin; DM: distal margin; LR: local recurrence; Ly: lymphatic invasion; TaTME: transanal total mesorectal excision; V: vascular invasion | | | | |
